# Hybrid system coupling ozonation and nanofiltration with functionalized catalytic ceramic membrane for ibuprofen removal

**DOI:** 10.1007/s11356-023-27225-5

**Published:** 2023-05-02

**Authors:** Kacper Szymański, Sylwia Mozia, Andre Ayral, Stephan Brosillon, Julie Mendret

**Affiliations:** 1 Department of Inorganic Chemical Technology and Environment Engineering, Faculty of Chemical Technology and Engineering, West Pomeranian University of Technology in Szczecin, Pułaskiego 10, 70-322 Szczecin, Poland; 2 Institut Européen des Membranes, IEM – UMR 5635, ENSCM, CNRS, Univ Montpellier, Place Eugène Bataillon, Montpellier, France

**Keywords:** Ozonation, Ceramic membrane, Nanofiltration, Ibuprofen, *Aliivibrio fischeri*, Toxicity

## Abstract

The investigations on the removal of ibuprofen (IBU) in a hybrid system coupling ozonation and nanofiltration with functionalized catalytic ceramic membrane are presented. The gaseous ozone into feed water in concentration of 11 g Nm^−3^ was supplied. Positive influence of catalytic ozonation on ibuprofen decomposition was observed. The application of catalytic nanofiltration membrane led to the ibuprofen removal of 91% after the first 15 min from the beginning of the O_3_/NF process, while at the same time, for the pristine membrane, it was equal to 76%. The investigations revealed incomplete degradation of drug under pH 3 after 2 h, i.e., 89%. On the other hand, the addition of inorganic salts did not affect the catalytic ibuprofen removal efficiency. Under acidic pH, the highest permeate flux decline (26%) was noted, whereas no differences between permeate flux measured under natural and alkaline conditions were observed. During the treatment process, three IBU by-products were detected, which significantly affected the permeate toxicity; however, after 2 h of catalytic nanofiltration, the product of treatment process was found as non-toxic.

## Introduction

The pollution of the aquatic environment by pharmaceuticals has become a serious problem. The discharge of brine wastewater containing pharmaceuticals degrades water quality and thus water cannot be directly used as potable water, even after desalination, and for industrial applications (Panagopoulos 2022). Conventional technologies applied in water or wastewater treatment plants are not efficient enough in drugs removal; therefore, there is an urgent need to develop new methods. Ozonation is an effective process for pharmaceutical degradation (Schmitt et al. 2020; Brillas 2022); however, it can generate smaller and more toxic by-products than the initial micropollutants. Hence, a promising solution could be the coupling of ozonation with membrane filtration, which allows for rejection of by-products and other molecules (e.g., colloids and ions) (Mansas et al. 2020a). Studies in literature survey related to pharmaceuticals elimination with application of both ozonation and membrane filtration are very limited. Most of the studies investigated ozonation or ozonation enhanced with H_2_O_2_ or UV as either effective pretreatment stage for removal of organics in membrane filtration feed stream or post-treatment stage to treat both effluent streams—permeate and retentate (Real et al. 2012; Byun et al. 2015; Miralles-Cuevas et al. 2017). For instance, Real et al. (Real et al. 2012) studied the efficiency of combined some chemical oxidation processes such as ozonation, chlorination, O_3_/H_2_O_2_, UV or UV/H_2_O_2_, and membrane separation, i.e., ultrafiltration (UF) or nanofiltration (NF) for removal of five PhACs (amoxicillin, hydrochlorothiazide, metoprolol, naproxen, and phenacetin) from various water matrices. They applied two approaches in their investigations. In the first case, the membrane processes were used as a pretreatment step, and effluents were subsequently treated by one of abovementioned chemical oxidation processes. In turn, the second approach involved application of chemical oxidation as a pretreatment stage before NF. The best removal efficiency of PhACs for pretreatment via NF followed by ozonation was found. In case of experiments conducted with natural water, the PhACs removal in the permeate reaching values higher than 97% at initial ozone dose of 2.25 mg L^−1^. When as a feed matrix a secondary effluent was applied, significantly higher initial ozone dose, i.e., 3.75 mg L^−1^, was needed to obtain the same effectiveness of pharmaceuticals removal. Treatment with using chlorination as post-treatment stage or UF pretreatment was characterized as less effective. In turn, chemical oxidation pretreatment followed by NF was much more effective for PhACs removal. In other studies (Byun et al. 2015), the effect of feed water pre-ozonation via reactions with molecular O_3_ or with radical species as primary oxidants on the permeate flux during NF of synthetic humic acid solution was investigated. Various combinations of pre-ozonation pH, calcium concentration, and O_3_ dosage were evaluated. According authors on the permeate flux strongly affected calcium concentration and ozone dosage rather than the ozonation mechanism. The researchers emphasized that fouling was mainly due to cake filtration and not pore blockage and partial mineralization of feed organics compounds via oxidation caused fouling mitigation. On the other hand, Miralles-Cuevas et al. (2017) stressed the role of understanding the degradation pathways leading to the formation of various degradation intermediates, since, based on the authors' research, more toxic products were produced during the treatment process than the starting compounds in the feed. The authors investigated the ozonation of NF retentates from real municipal wastewater treatment plant in terms of microcontaminants removal and toxicity. Treatment of NF rejection needed 2.75–4.5 g O_3_ m^−3^, while 4.5 g O_3_ m^−3^ was less than 50% of the ozone required for direct treatment of effluent.

The work of Ouali et al. (2022) describes hybrid ozonation/NF process enhanced with H_2_O_2_ for treatment of drinking and river water enriched with pharmaceuticals (carbamazepine and sulfamethoxazole). Nevertheless, in this system, flat sheet organic polyethersulfone and polyamide, not ceramic NF membranes, were applied. Moreover, they were not catalytic membranes. In the literature, several processes which employed UF or microfiltration (MF) catalytic ceramic membranes were investigated (Karnik et al. 2005; Park et al. 2012; Zhu et al. 2012; Wang et al. 2013; Mei et al. 2015). These studies mostly focused on removal of humic substances (Park et al. 2012; Zhu et al. 2012; Wang et al. 2013; Mei et al. 2015) or trihalomethanes (Karnik et al. 2005; Wang et al. 2013). Regardless of the membrane type, the ozonation can be performed before membrane filtration or it can be enhanced by membrane filtration, where the feed water and ozone are directly injected into the membrane area. However, there are no literature reports regarding the hybrid systems utilizing catalytic ozonation and membrane separation (nanofiltration), which proves the novelty of the presented investigations. In addition, the cited works did not carry out toxicity analyses of the purified solutions, which were presented in this article. In this context, the objective of the research is to investigate the possibility of removal of a model pharmaceutical—ibuprofen (IBU), being a representative of non-steroidal anti-inflammatory drugs (NSAIDs), with application of the hybrid process coupling NF and catalytic ozonation. The NF is a promising separation method, which could reject small organic contaminants (200 Da), whereas O_3_ was used to decompose the harmful pharmaceutical. In the experiments, a tubular catalytic ceramic membrane was applied, which is ozone-resistant, in combination with ozonation let for achieving a high permeate flux without membrane damage in opposite to polymeric ones (Karnik et al. 2005). The ozonation of the feed water and membrane filtration was simultaneously performed. By the coupling of ozonation with the action of the catalyst deposited onto NF membrane, an enhanced production of hydroxyl radicals could be obtained and, in result, the degradation of IBU was improved. During the research the effectiveness of the treatment process was evaluated based on changes of ibuprofen concentration in time. Moreover, the use of ozonation can be beneficial as it could reduce the NF membrane fouling due to the strong oxidative properties of ozone, what has significant impact on the practical application of this technology in a full scale. The membrane performance was analyzed during the study based on changes of permeate flux in time as well. Since, the intermediates products of ozonation could exhibit in some case higher toxicity than the initial contaminants (Gomes et al. 2017), the monitoring of the toxicity not only of the treated feed water but the produced permeate as well is of high importance. Hence, the standardized acute toxicity tests were performed with application of bacteria *Aliivibrio fischeri* in order to evaluate possible toxicological effect of feed and permeate, what further emphasizes the novelty of the presented research.

Ibuprofen was selected for the investigations as a representative of non-steroidal anti-inflammatory drugs. It is characterized by carcinogenic and non-steroidal endocrine disrupting drug with harmful effects over fungal, bacterial, algae, microorganisms, crustaceans, and fishes and can be potentially hazard for human health (Brillas 2022). It is derived from propionic acid, and a broad spectrum of action, making it one of the most consumed drugs worldwide (Almeida et al. 2022). The pharmaceutical is widely found in the aquatic environment. Due to its complex degradability, several intermediates formed during its decomposition are not completely removed by conventional methods treatment (Almeida et al. 2022).

In the present investigation, the hybrid system coupling ozonation and NF with catalytic ceramic membrane for ibuprofen removal were proposed. Especially, the effect of pH and inorganic salts on the membrane performance and effectiveness of treatment process was determined. Additionally, the ecotoxicity tests of treated solutions were conducted.

## Experimental

### Chemicals

Ibuprofen (IBU) (C_13_H_18_O_2_, 206,28 g mol^−1^) and inorganic salts (MgSO_4_·7H_2_O, NaHCO_3_, NaNO_3_, NaH_2_PO_4_·2H_2_O, CaCl_2_) were obtained from Sigma Aldrich, USA. The concentrations of the salts applied in the experiments were selected based on the literature (Nawrocki and Kasprzyk-Hordern 2010; Szymański et al. 2016; Khuntia et al. 2016) in order to evaluate the influence of typical inorganic compounds present in natural waters.

### Feed

During the experiments, 10 mg L^−1^ of ibuprofen was applied. In the experiments with salts, the proper amount of inorganic salts (Table 1) was added into distilled water and mixed thoroughly.

**Table 1:** Concentration of inorganic salts applied in the experiments

Compound	[mg L^−1^]
MgSO_4_·7H_2_O	769 ± 2
NaHCO_3_	420 ± 1
NaNO_3_	3 ± 0.2
NaH_2_PO_4_·2H_2_O	8 ± 0.2
CaCl_2_	111 ± 2

### Membrane

One channel asymmetric tubular ceramic membrane with outer/inner (*d*_*o*_/*d*_*i*_) diameter of 10/7 mm and length of 250 mm was applied (IKTS, Germany). The membrane had an effective filtration area of 5.5·10^–3^ m^2^ and molecular weight cut off (MWCO) of 200 Da (according to the manufacturer). The support of the membrane is made of α-Al_2_O_3_, and the microporous separation layer is based on TiO_2_.

This commercial ceramic nanofilter was functionalized by depositing an additional mesoporous layer made of iron oxide (thickness of ~ 80 nm) on its microporous separative layer (thickness of ~ 100 nm). Synthesis conditions as well as physicochemical characteristics and catalytic efficiency of such functionalized membranes are detailed in a previous paper (Mansas et al. 2020b).

### The installation set-up and process conditions

**Fig. 1:**
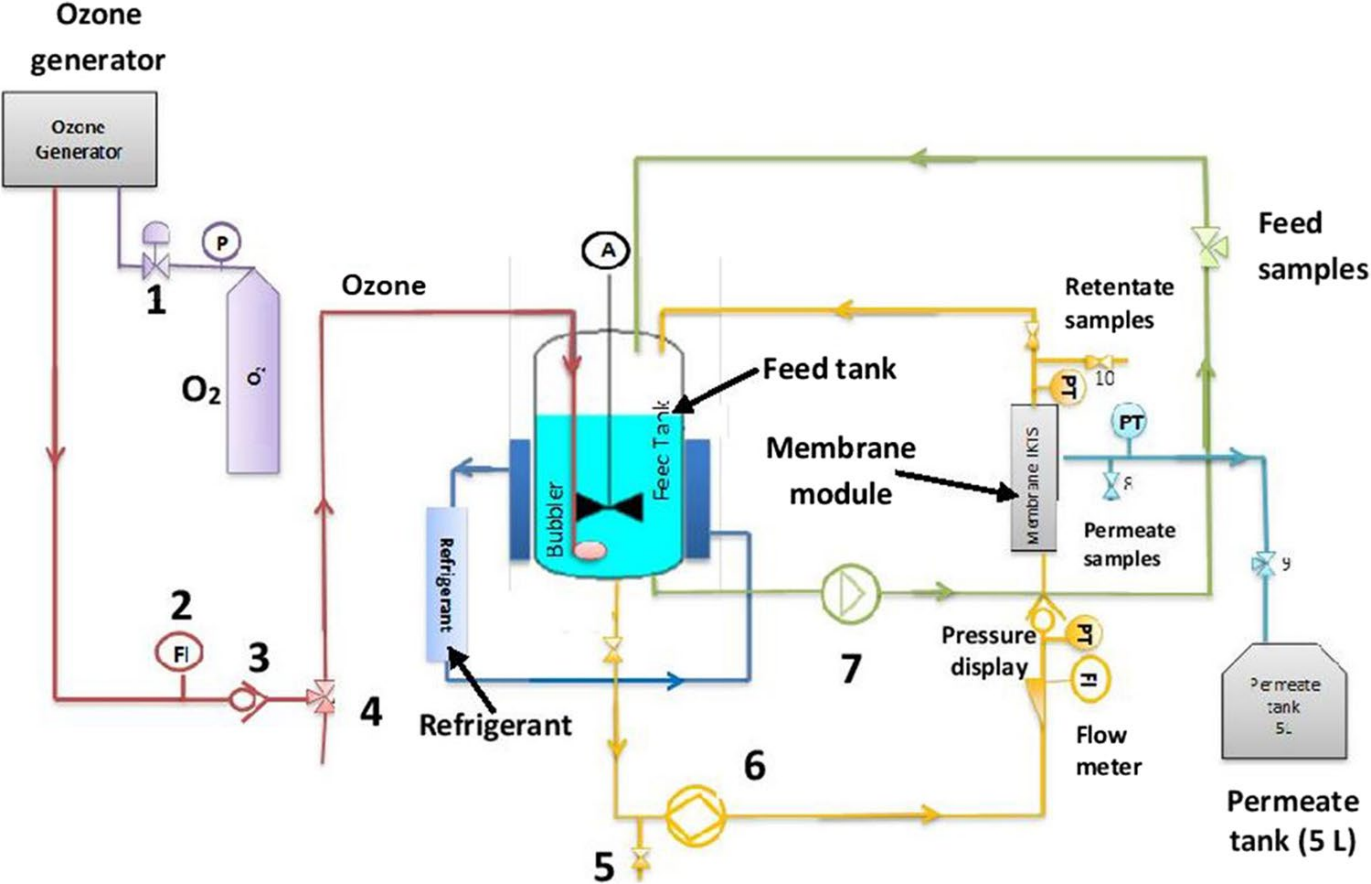
Schematic diagram of the installation applied in the experiments. 1-Overflow, 2-Brooks flow meter, 3-check valve, 4-three-way valve, 5-drain valve, 6,7-pumps, 8,9,10-valves

The experiments were carried out in a laboratory scale installation, which scheme is presented in the Fig. 1

The membrane was inserted in a stainless-steel module and was held in a vertical position with clamps at its two ends. The total volume of the feed circulated in the system at the beginning of the process was 4 L. The feed was pumped in the loop using a positive displacement pump at a flow rate of 69 L h^−1^ with a tangential speed of 0.5 m s^−1^. The transmembrane pressure was 10 bar and the temperature of the feed was kept at 20 °C. The gas flow rate of O_3_ injected in the tank reactor was 20 L h^−1^ and the concentration of the injected gaseous ozone was 11 g Nm^−3^. Before opening the permeate valve and starting ozonation, the feed circulated in the system for 2 h up to reaching the stable concentration of IBU (adsorption of ibuprofen onto membrane) was performed. During the experiments, the weighting of the collected permeate during 2 h was performed and permeate flux after determined time intervals was evaluated. After each experiment, the membrane was rinsed with pure water, cleaned by filtration of ozonized water during 40 min, and again rinsed with distilled water. The permeability of the pristine and functionalized membranes with O_3_ as well as without ozone was measured. The pH was corrected to a value of 3 with using HCl. While after adding inorganic salts to the water, it was 8.5. In this case, no correction of pH was needed.

The summary of experiments carried out during the research has been presented in the Table 2.

**Table 2:** The experiments carried out during the investigations on ibuprofen removal

Experiment	Process description
Pristine, NF	pH 6.5, process time: 2 h, IBU concentration: 10 mg L^−1^
Pristine, O_3_/NF	pH 6.5, process time: 2 h, IBU concentration: 10 mg L^−1^, injected gaseous ozone: 11 g Nm^−3^
Functionalized, NF	pH 6.5, process time: 2 h, IBU concentration: 10 mg L^−1^
Functionalized, O_3_/NF	pH 6.5, process time: 2 h, IBU concentration: 10 mg L^−1^, injected gaseous ozone: 11 g Nm^−3^
Functionalized, O_3_/NF_pH 3	pH 3, process time: 2 h, presence of salts: MgSO_4_·7H_2_O, NaHCO_3_, NaNO_3_, NaH_2_PO_4_·2H_2_O, CaCl_2_, IBU concentration: 10 mg L^−1^, injected gaseous ozone: 11 g Nm^−3^
Functionalized, O_3_/NF_pH 8.5	pH 8.5, process time: 2 h, presence of salts: MgSO_4_·7H_2_O, NaHCO_3_, NaNO_3_, NaH_2_PO_4_·2H_2_O, CaCl_2_, IBU concentration: 10 mg L^−1^, injected gaseous ozone: 11 g Nm^−3^

In the first stage of the research, the reference experiments with using pristine membrane and in the presence or in the absence of ozone were conducted (Table 2). No salts were added during these processes. In case of second stage of the studies, the experiments with application of functionalized catalytic membrane were performed (Table 2). Herein, the processes with or without ozonation were carried out as well. Moreover, the influence of salts and pH during hybrid ozonation-NF processes was evaluated (Table 2).

### Analytical methods

The concentration of ibuprofen and presence of by-products were determined using the high-performance liquid chromatography coupled with two mass spectrometers (LC/MS/MS) using an e2695 apparatus from Waters Alliance and mass spectrometers of Quattro Micro and PDA 996 types. The involved equipment was as follows: a Waters 2695 pump, an autosampler with a 20 μl loop, a Waters 2695 separation module (HPLC), and a Waters Micromass (Wythenshawe, Manchester, UK) a Quattro Micro mass spectrometer (MS) equipped with a Electro Spray ionization (ESI) probe in negative mode. A column (C18 Waters HSS-T3: 100 mm * 2.1 mm, 3.5 μm particle size) was used with a buffer A (95% LC grade water + 5% LC grade acetonitrile + 0.1% formic acid) and a buffer B (100% LC grade acetonitrile + 0.1% formic acid). For ozone analyses, the indigo method was used (Bader 1982). It is based on the decolorization of the indigo reagent by ozone. The absorbance at 600 nm was measured using a UV–VIS spectrometer (Jenway 7315). The pH was monitored with using pH meter (Thermo Fisher Scientific). Conductivity was determined using Ultrameter™ 6P (MYRON L COMPANY, USA).

### Toxicity measurements

The toxicity of feed and permeate samples was evaluated using the Microtox® LX system (Modern Water, USA). In the Microtox® toxicity test, marine bacteria *Aliivibrio fischeri* for evaluating the toxicity of substances are applied. The test is based on the decreasing of bioluminescence of bacteria after exposure to the toxic factors (Ngwoke et al. 2021). In brief, the idea of the test is as follows: (1) freeze dried cultures of *A. fischeri* were reconstituted and their luminescence was measured, (2) then the bacterial culture was gently mixed with a sample and incubated for 15 min, (3) the luminescence was read after 5 min and again after 15 min, and (4) the changes of luminescence intensity were given as a nominal % change value compared to the luminescence measured in the control sample.

## Results and discussion

### Permeability of the membranes

**Fig. 2:**
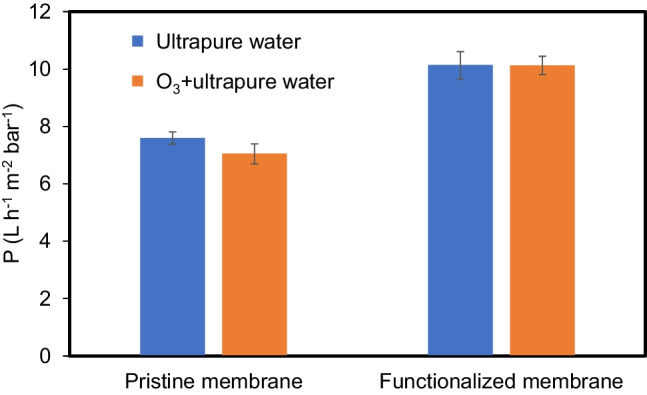
Permeability of the pristine and functionalized membranes for pure water and with ozone application

The water permeability of the both ceramic pristine and functionalized NF membranes was determined to be 7.6 and 10.1 L m^-2^ h^-1^ bar^-1^, respectively, for pure water. During application O_3_, the permeability of pristine membrane slightly decreased to value 7.1 L m^-2^ h^-1^ bar^-1^, and in case of catalytic membrane, there was no influence of ozone (Fig. 2). The observed results could be related to the ozone consumption during the crossing through the catalytic layer of the functionalized membrane (Mansas et al. 2020b). On the other hand, the presence of ozone nanobubbles positioned indirectly next to the micropores of the TiO_2_ layer of pristine membrane could cause pores blockage and, in results, the decreasing of permeance (Fig. 2).

### Ibuprofen removal

**Fig. 3:**
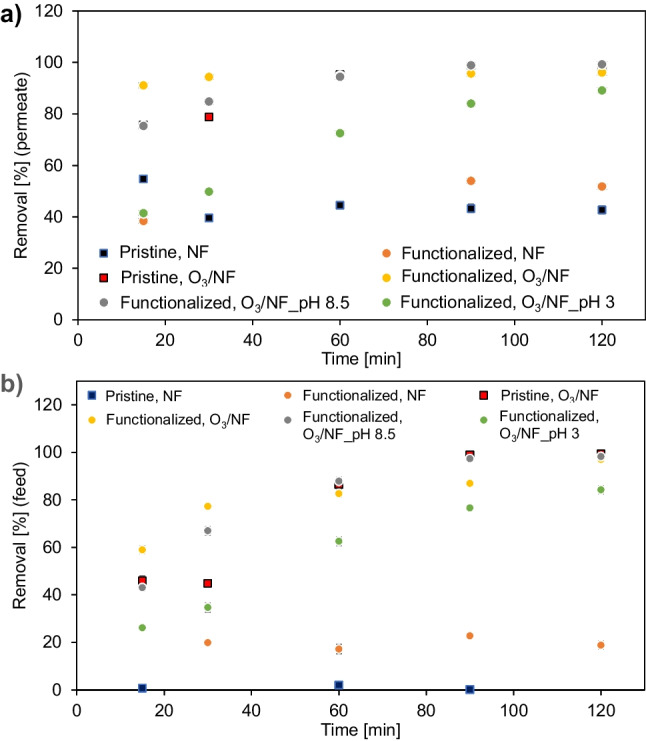
Effect of feed composition on the ibuprofen removal in the (**a**) permeate and (**b**) feed. Initial concentration of ibuprofen in the feed: 10 mg L^−1^, transmembrane pressure: 10 bar

Before each NF process, the ibuprofen adsorption step (NF carried out with closed permeate valve) was carried out for 2 hours. After this time, the permeate valve was opened and, additionally, in the case of processes with ozone, the ozone generator was switched on. Then, for the next 2 hours of the process, the removal of the model pharmaceutical in the feed and permeate was assessed. The functionalized membrane was used in the experiments at different pH. The results are presented in the Fig. 3 for various feed characteristics.

As a comparison, the NF processes were performed using both a pristine and a catalytic membrane. In this case, the pristine membrane separated the ibuprofen for about 40%, and the functionalized one, for more than 50% (Fig. 3a). The application of ozone led to remove the pharmaceutical after 90 min of the O_3_/NF process in more than 90%, while after two hours of the process, almost complete removal of ibuprofen was noted, both for the pristine and catalytic membranes (Fig. 3a). It should be also emphasized that in the case of the catalytic membrane, the ibuprofen removal reached the result of 91% after the first 15 min from the start of the O_3_/NF process, while at the same time for the pristine membrane, it was equal to 76% (Fig. 3a).

In the next stage of the research, the influence of the presence of inorganic salts in the feed and pH was analyzed. In the presence of salt and pH 3, the removal of the model pharmaceutical was significantly slower from the beginning of the process. After 15 min, it was 41% solely, and after 2 h of the process, it was 89% using functionalized membrane. In turn, at pH 8.5, after the first 15 min of the experiment, the removal of IBU was almost two times higher (75%), and after 90 min, it reached the value of 99% (Fig. 3a).

Considering the ibuprofen removal results in the feed (Fig. 3b), a similar removal trend for this pharmaceutical can be seen. Nevertheless, it should be emphasized that in the previous case, the removal of IBU in the permeate was influenced by ozone and the membrane, while in the feed, only by ozonation. Based on the results shown in the Fig. 3a and b, it can be concluded that the main role in the ibuprofen removal was due to the ozonation process; however, membrane separation contributed to the quite high overall treatment efficiency.

**Fig. 4:**
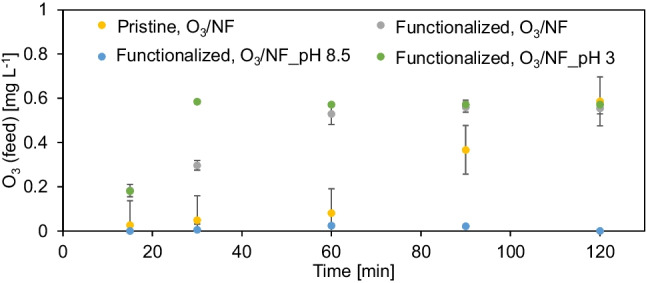
Changes of dissolved ozone concentration in the feed during the process. Operation conditions: continuous ozonation, ozone gas concentration 11 g Nm^−3^

Since the decomposition of ozone in solution to form OH radicals is highly pH dependent, the present results could find the explanation in the O_3_ evolution during the process (Fig. 4). It is established that there is strong correlation between ozone decomposition in water and pH—it occurs faster with an increase of pH (Nawrocki and Kasprzyk-Hordern 2010). No changes of pH after adjusting the pH to 3 or 8.5 during the process were noted. In case of catalytic ozonation-NF experiment (O_3_/NF), the pH was 6.5 and maintained constant during 2 h.

Generally, alkaline conditions during ozone oxidation of organic compounds are in favor of the indirect hydroxyl radicals forming (oxidation potential 2.80 V) and could be beneficial to the degradation of targets than acidic conditions (O_3_ oxidation potential 2.07 V) (Miao et al. 2015), what is reflected in the present investigations. When the pH increases, the IBU decomposition enhancement is observed, since the larger amount of OH radicals is generating, due to more OH anions forming on the catalytic membrane surface (Ejhieh and Khorsandi 2010). Taking into account the results in the Fig. 4, it can be seen that the lowest O_3_ concentration during the whole process carried out in the presence of salts under alkaline conditions was noted. Considering the abovementioned ibuprofen removal results, it can be concluded that from the first minutes of the experiment, ozone was consumed for IBU degradation (Fig. 3b) under these conditions. On the other hand, in the case of acidic pH, ozone consumption was significantly slower (Fig. 4). It means, some small amount of O_3_ was used for hydroxyl radicals production. In turn, for catalytic membrane, a fast increasing of the ozone in time was observed, due to high transfer of ozone to OH radicals and IBU decomposition. A significantly slower ozone concentration increasing in case of pristine membrane was caused by lower amount of hydroxyl radicals forming and not so fast using them for oxidation of pharmaceutical. Catalytic ozonation, contrary to application of ozonation solely, enables the formation of hydroxyl radicals also at a low pH (Nawrocki and Kasprzyk-Hordern 2010).

### By-products detected during the IBU degradation

**Fig. 5:**
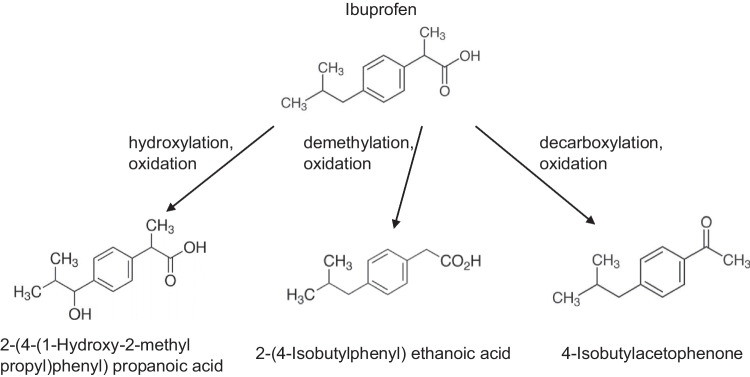
Ozonation pathways and by-products of ibuprofen during hybrid O_3_/NF process

Reactions of ozone with ibuprofen led to formation of by-products and among them the most probable are ketones, aldehydes, and carboxylic acids (Ikhlaq et al. 2015). A high number of papers have reported the generation of some by-products of IBU upon the action of the OH radicals generated during ozonation (Michael et al. 2014; Saeid et al. 2020; Huang et al. 2021; Brillas 2022; Krakstrom et al. 2022). Three parallel oxidation pathways after initial degradation of ibuprofen by **·**OH are possible: hydroxylation, demethylation, and decarboxylation (Fig. 5) (Brillas 2022). Additionally, they can interact with each other and create various by-products (Brillas 2022). The obtained results revealed three intermediates, i.e., 2-(4-(1-Hydroxy-2-methyl propyl)phenyl) propanoic acid formed during hydroxylation and further oxidation (Saeid et al. 2020), 2-(4-isobutylphenyl) ethanoic acid (demethylation and further oxidation) (Michael et al. 2014), and 4-isobutylacetophenone in result of decarboxylation and further oxidation (Fig. 5) (Huang et al. 2021). The molecular masses of the identified by-products are as follows: 221.12 Da, 193.12 Da, and 177.13 Da, respectively.

Determination of intermediate decomposition products is very important from practical point of view, since the oxidation of organic compounds in many cases led to formation more complex and toxic compounds than the initial pollutants (Miralles-Cuevas et al. 2017). These compounds can reduce the efficiency of the NF process and/or affect the toxicity of the permeate (Miralles-Cuevas et al. 2017). These issues are discussed in the further paragraphs.

### Membrane performance during IBU removal

**Fig. 6:**
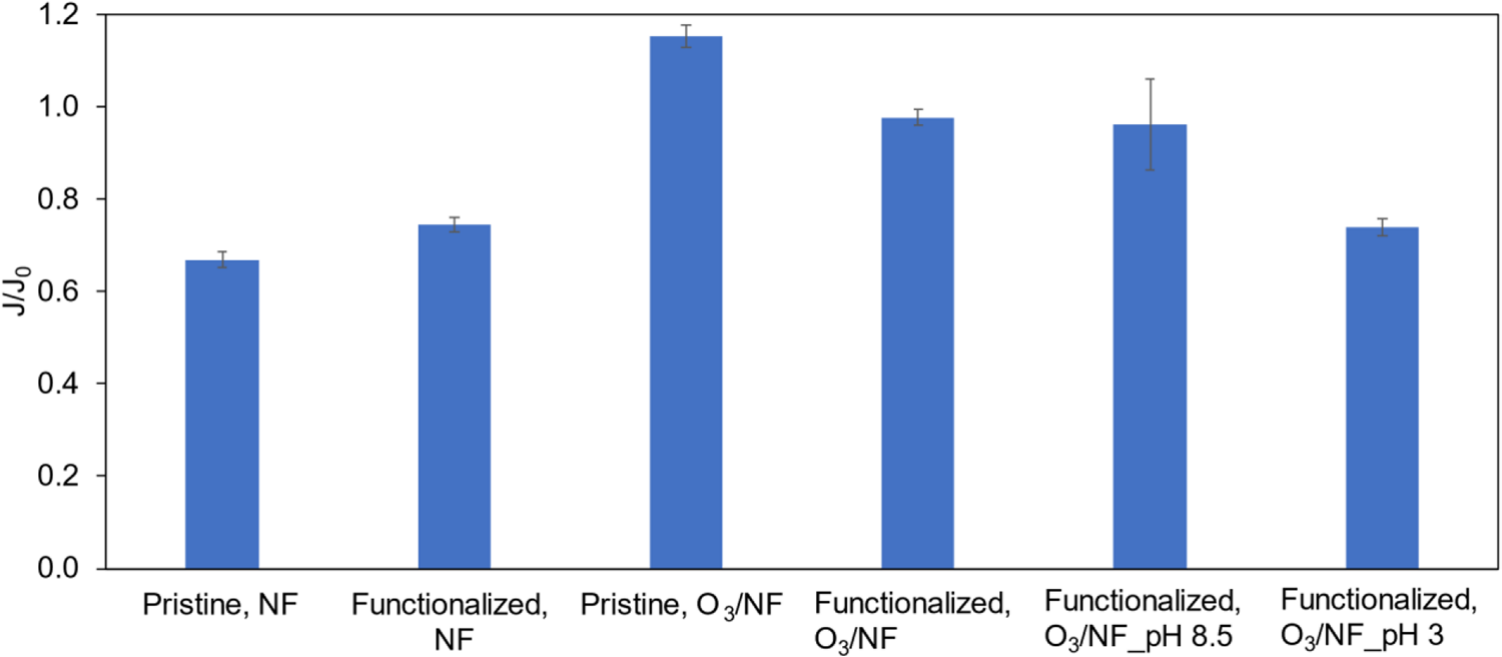
Normalized permeate flux decline after 2 h of removal of the ibuprofen depending on the feed composition and pH

The idea of proposed system, i.e., the hybrid ozonation-NF process, was the degradation of ibuprofen by ozonation and the separation of the products decomposition by the membrane in order to obtain a purified product (permeate). Nevertheless, intermediates formed during the process and undecomposed IBU could effectively block the pores of the membrane; thus, reducing the efficiency of the filtration process was observed (Karnik et al. 2005). Figure 6 presents the normalized permeate flux decline after 2 h of removal of the model PhAC depending on the feed composition and pH.

The largest decrease of the permeate flux was measured in the case of NF with the using of pristine membrane, probably due to blocking the pores of the membrane by IBU molecules presented in the feed. Interestingly, a slight increase in the permeate flux was also observed for this membrane during ozonation. It could be caused by the electrostatic interaction between the PhAC molecules and the hydrophilic surface of the pristine membrane, which separation layer was made of TiO_2_ (Zhao et al. 2018; Hossain et al. 2019). On the other hand, in the case of NF of the ibuprofen solution through a functionalized membrane, a decrease in the permeate flux by 25% after 2 h of the process was observed. During catalytic ozonation, the flux practically did not decrease (only 2% after 2 h) (Fig. 6), which was reflected in the decomposition of ibuprofen from the beginning of the experiment (Fig. 3b). A slight decrease could have been influenced by the residual undecomposed ibuprofen or the intermediate products of catalytic ozonation. Since the catalyst was coated on ceramic membrane for simultaneous proceeding of catalytic ozonation and membrane separation process, the membrane foulants could be timely decomposed before their accumulation on the membrane surface or within its pores (Zhang et al. 2016). In the presence of salt (pH 8.5), the decrease of the permeate flux was negligible as well (4% after 2 h). On the other hand, during the removal of ibuprofen at the feed pH of 3, a 26% decrease in the permeate flux was noted after 2 h of the experiment (Fig. 6). These results were also reflected in the degree of decomposition of the model PhAC. Moreover, the obtained results may also be the result of electrostatic interactions between ibuprofen molecules and the membrane surface. It is well established that the pH of the solution is a crucial parameter which has a significant effect on the ·OH formation and organic pollutant properties related to decomposition (Dalrymple et al. 2007). Since the pH was adjusted at 8.5, the IBU degradation increased, due to the formation higher amount of OH radicals, whereas the reverse situation occurred for pH 3 (Kezzim et al. 2017). And this, in turn, was reflected into the values of the observed permeate flux.

It is worth noting that the membrane did not reject inorganic salts presented in the feed due to their lower molecular weights than MWCO of NF membrane applied in the experiments (200 Da). Initial conductivity of the feed solution was about 1720–1732 μS cm^−1^ for samples with inorganic salts both at pH 3 and 8.5. After 2 h of process in the permeate conductivity varied from 1918 to 2215 μS cm^−1^ and in the feed was 1952–2287 μS cm^−1^. The higher value of the feed and permeate conductivity in relation to the treated solution was most likely due to the presence of intermediate products of ibuprofen decomposition. Such low salts rejection, expressed in a high conductivity, by ceramic NF membrane was obtained as well by Fujioka et al. (Fujioka et al. 2018).

### Ecotoxicity study during IBU removal

Toxicity testing is a very important method to evaluate the effectiveness of treatment technologies. The solutions exhibit different toxicity effects expressed by microorganisms mortality rate. The Microtox® test applied in this experiment is a well-established test measuring the acute toxic impact on the bacteria *Aliivibrio fischeri*. The exposure time of the bacteria with the samples was 15 min and the luminescence analysis was then performed. The toxicity evolution was expressed in terms of percentage of luminescence inhibition. The samples for which the results are less than 20% are considered as non-toxic, between 20 and 50% as low-toxic, and above 50% as toxic (Persoone et al. 2003). In case of the treated solutions under acidic pH and in the presence of inorganic salts, all samples were characterized as toxic regardless of process time (99.99% of toxicity) (data not presented). The obtained results were caused by such low pH. The most common stressor, which had an impact on the microorganisms, is high osmotic pressure of the feed. In the acidic environment, the wall cell of bacteria was disrupted and bacteria cell died, what affected on the observed toxicity.

**Fig. 7:**
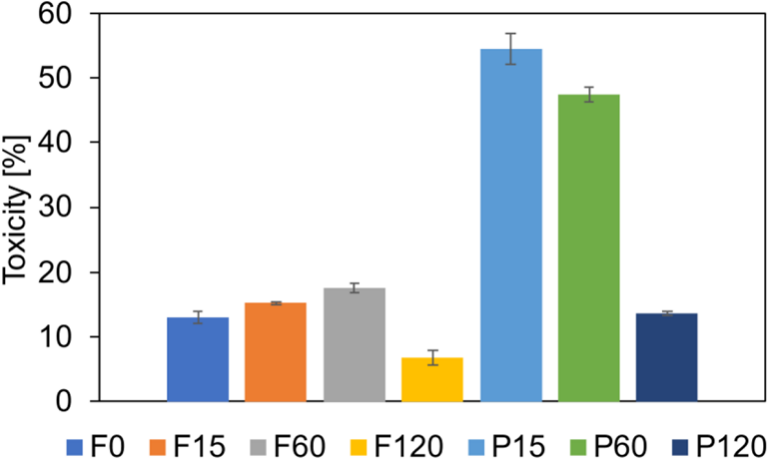
Toxicity of treated solution during hybrid catalytic ozonation-NF process under pH 8.5 and in the presence of inorganic salts. F0—initial feed, F15, F60, F120—feed samples collected after 15, 60 and 120 min of the process, P15, P60, P120—permeate samples collected after 15, 60 and 120 min of the process

In turn, in the present of salts under alkaline conditions, the initial feed did not exhibit toxicity (13%) (Fig. 7). During the O_3_/NF process, some increasing of toxicity of treated feed was noted; nevertheless, the samples still were non-toxic. After 15 and 60 min, the toxicity was 15 and 18%, respectively. Finally, at the end of the process toxicity of F120 sample was two times lower than F0, i.e., 7 vs. 13% (Fig. 7). Opposite situation for permeates was noted. The permeate after 15 min exhibited high toxicity (54%). The applied treatment led to decrease of the permeate toxicity to about 47% after 60 min of the experiment (Fig. 7). These results indicate that the presence of the other feed components, i.e., by-products, which passed through NF membrane affected the toxicity values (Quero-Pastor et al. 2014; Miralles-Cuevas et al. 2017). The course of changes of mortality suggests that although the efficiency of degradation was high (Fig. 3b), the formation of by-products of oxidation was still faster than their complete removal. Finally, at the end of the process treatment, the permeate (product of treatment process) was characterized as non-toxic. The toxicity was the same like in case of the initial feed solution. The ecotoxicity of IBU (10 mg L^−1^) toward alga *Selenastrum capricornium* cultures and for optimal pH (8.5–9) and stirring of the system for 20 min during ozonation was studied by Quero-Pastor et al. (Quero-Pastor et al. 2014). No toxic effect of PhAC on the model organism was noted at the beginning of the treatment process. However, after the stirring and pH adjustment, the percentage inhibition visible increased, in results of formation of hydroxy-ibuprofen metabolite. Moreover, at concentration of IBU 10 mg L^−1^, a higher toxicity due to oxidative process proceeding was found (Quero-Pastor et al. 2014).

### Economic aspects of IBU removal in the O_3_/NF system

The cost of proposed treatment process is mainly related to the energy of ozone generation and NF. Nevertheless, it should be emphasized that the costs of novel treatment technologies depend mostly on the type of process and design configuration proposed; therefore, it is difficult to compare the costs of various technologies properly. For instance, the application of O_3_ at low doses is economically effective and less expensive than granular activated carbon (GAC) (Pistocchi et al. 2022). The ozonation is considered as a promising method for increasing the efficiency in microbial load and pharmaceutical reductions with moderate treatment costs (Zagklis and Bampos 2022). For ozonation systems, it was reported that the cost of treatment of 1 m^3^ of secondary effluent is 0.03 EUR (Chys et al. 2018, Zagklis and Bampos 2022). According to Choubert et al. (2017) in France, it is 0.1–0.2 EUR m^−3^ depending on the size of the plant, operating conditions, and the supply chain of reagents. In some publication, it was reported that the energy consumption (kWh) of catalytic ozonation and filtration in the cross flow system for Lake Lansing water was 3.032*10^–3^ and 6.2*10^–4^, respectively, calculated for 1000 L of treated water with O_3_ dose of 20 μg s^−1^ (Wang et al. 2017). Taking into the calculation the price of 1 kWh as 0.2126 EUR (mean price in Europe), the cost of such system is 0.0008 EUR m^−3^. Assuming the energy consumption of 0.3 kWh m^−3^ of treated water in case of ozonation and 1 kWh m^−3^ for NF (Rizzo et al. 2019), the presented hybrid O_3_/NF system required 0.0011 EUR m^−3^. This cost is lower than the values reported in the abovementioned literature (Choubert et al. 2017; Chys et al. 2018; Zagklis and Bampos 2022); however, it must be noted that the system was not optimized and the investigations were carried out in the laboratory scale installation. Such low value could also result from using functionalized catalytic membrane, which significantly improved decomposition efficiency (Pistocchi et al. 2022). For comparison, Zagklis and Bampos (2022) stated that chlorination and UV irradiation are characterized by the lowest treatment costs (0.004 EUR m^−3^). The cost of photocatalytical TiO_2_/UV treatment of 1 m^3^ of water containing 0.8 mg L^−1^ total organic carbon was 0.44 USD, whereas in case of 8.0 mg L^−1^ total organic carbon, it was 0.81 USD (Basile et al. 2018). On the other hand, the cost of phenol degradation, calculated on a basis of degradation rate constants, was significantly higher, i.e., 2285 USD m^−3^, than that of trichloroethylene degradation (3.99 USD m^−3^) (Basile et al. 2018). In case of membrane bioreactors (MBR), the treatment cost varied from 0.10 to 0.68 USD m^−3^ (Gao et al. 2022). A ketoprofen decomposition in a submerged photocatalytic membrane reactor generated cost of 13–24.4 USD m^−3^ order^−1^, depending on applied conditions (Szymański and Mozia 2023).

### A comparison of proposed system with other techniques dedicated to IBU removal

The literature survey reported other techniques dedicated to IBU removal. Among them, there are photolysis (Yan and Song 2014), non-thermal plasma treatment (Zeng et al. 2015), UV/H_2_O_2_ (Liu et al. 2017) and UV/O_3_ (Mehrjouei et al. 2020) hybrid processes, Fenton (Zhang et al. 2019), membrane separation processes (Ganiyu et al. 2015; Arefi-Oskoui et al. 2019), and adsorption (Silva et al. 2023). It was found that the application of single non-thermal plasma treatment led to quite fast IBU degradation (91.7% in 80 min); however, the mineralization was very low (ca. 30%) (Zeng et al. 2015). In general, the main drawback of such technology is low ability to mineralize ibuprofen and its by-products. Shu et al. (2013) studied the degradation of IBU in concentration of 10–40 mg L^−1^ with addition of 25 or 50 mg L^−1^ H_2_O_2_ at neutral pH in the reactor equipped with an inner Hg lamp (200–320 nm). By increasing of hydrogen peroxide concentration, the ibuprofen removal was 1.6-fold greater. Nevertheless, H_2_O_2_ due to its strong reactive potential creates a high toxicity of treated solution (Szymański et al. 2018). A hybrid O_3_/H_2_O_2_ system is the most widely investigated among hybrid ozonation techniques, despite high toxicity of reagents (Mehrjouei et al. 2020). It has been applied for pure and natural waters, hospital wastewater, and wastewater treatment plant effluents (Brillas 2022). The first recognized research involving O_3_/H_2_O_2_ system for IBU removal is work of Zwiener and Frimmel (2000). They treated 2 μg L^−1^ of ibuprofen in pure water and river water (total organic carbon concentration was 3.7 mg L^−1^) and pH 7.5 by application of 0.4–1.8 mg L^−1^ of H_2_O_2_ and tank reactor under continuous supply of 1–5 mg O_3_ L^−1^. After 10 min of ozonation, 99% of ibuprofen removal was achieved for 1.8 mg H_2_O_2_ L^−1^ and 5 mg O_3_ L^−1^. In order to remove ibuprofen, Fenton-based processes were applied as well. However, they have two main drawbacks, i.e., process optimum at pH near 3 and generation of a large amount of sludge rich in iron hydroxides, which requires post-treatment step (Chehrenegar et al. 2016). An interesting and novel technique of IBU removal based on adsorption has been proposed by Silva et al. (Silva et al. 2023). External magnetic field caused the adsorption of IBU on magnetic beads of alginate/polypyrrole/ZnFe_2_O_4_ (Alg/PPy/ZnFe_2_O_4_) in the amount of 108.2 mg g^−1^ in 70 min. Despite high efficiency of this method, there is a necessity of adsorbent regeneration. Our research group investigated a photocatalytic membrane reactor utilizing membrane distillation, equipped with capillary polypropylene membrane in order to remove the ibuprofen, naproxen and diclofenac from water or wastewater (Mozia et al. 2016). It was found that the efficiency of the treatment process was strictly dependent on the feed composition, and the key factors that influenced the photodegradation of the IBU were inorganic ions, organic compounds, and turbidity.

The method described in this article is undoubtedly very effective for removing ibuprofen (99% after 15 min) and obtaining a non-toxic permeate. In addition, the simultaneous conducting of catalytic ozonation and NF significantly reduces the time needed for its removal. Nevertheless, tests with a real matrix are required.

All applied treatment technologies depend on the feed matrix and required degree of purification. Then, the type of process and design configuration are proposed. Hence, the comparison of the technologies and clear indication of the best method for ibuprofen removal is complex.

## Conclusions

A removal of ibuprofen in a hybrid ozonation-NF system equipped with catalytic ceramic membrane was investigated. The permeability of the pristine and functionalized membranes was determined to be 7.6 and 10.1 L m^−2^ h^−1^ bar^−1^, respectively, for pure water. The application of ozone caused slight decrease of pristine membrane permeability to value 7.1 L m^−2^ h^−1^ bar^−1^, and for catalytic membrane, no influence of O_3_ was observed. It was found that high effectiveness of decomposition of pharmaceutical occurred even in the present of inorganic salts and pH 8.5. During the first 15 min of the experiment, the removal of IBU was equal to 75%, and after 90 min, it reached the value of 99%. Significantly lower degradation of ibuprofen under acidic pH was observed, i.e., 41% and 89%, after 15 min and 120 min, respectively. This was associated with the low amount of OH radicals formed from ozone. The highest permeate flux decline was noted in the presence of salts and at pH 3 as well (26% after 2 h of process). Under alkaline conditions and in the absence of salts, the flux decreasing was negligible due to fast decomposition of ibuprofen (4% and 2%, respectively). In case of the process with inorganic salts and under pH 8.5, the O_3_ consumption was the highest during whole time. Three by-products, i.e., 2-(4-(1-Hydroxy-2-methyl propyl)phenyl) propanoic acid, 2-(4-isobutylphenyl) ethanoic acid, and 4-isobutylacetophenone were detected during ozonation, which had an influence on the acute toxicity toward *Aliivibrio fischeri*. The initial feed was assigned as non-toxic. The applied treatment resulted in an increase of toxicity in the case of permeate, which was attributed to the formation of more intermediates, which passed through the membrane upon NF. Nonetheless, it was found that the product of O_3_/NF process (i.e., permeate) was non-toxic at the end of the process (after 2 h). Finally, it can be concluded that the hybrid ozonation-NF system thanks to synergistic effects induced by contaminant oxidation and rejection using catalytic ceramic membrane showed excellent performance toward the elimination of ibuprofen, promoting its efficient removal (98%) and also decreasing its toxicity. This configuration will be tested with a real wastewater treatment plant effluent so as to deeply investigate the beneficial effect of this coupling on fouling dynamics.

The results in the present manuscript demonstrated that proposed O_3_/NF system can be considered as useful for ibuprofen removal. Nevertheless, further studies are still needed before taking advantage of their potentiality at industrial level. First of all, the investigations with application of real matrices should be considered and economical assessment of proposed system in a pilot plant scale should be carried out. Coupling of catalytic ozonation and NF process is a promising solution, due to the potential rejection of small molecules and fast degradation of organic compounds, including by-products, with a very good membrane performance. Despite the fact that ceramic membranes are more expensive than organic ones, they are characterized by high potential for catalytic ozonation, since they exhibit strong resistance to ozone and a catalytic layer could be easily deposited on their surface. Future work should be focused as well on finding the optimal conditions of the process in order to reduce the treatment costs, especially related to the using of low amount of ozone. It could be obtained, e.g., by application of the ozonation contactor.

## Data Availability

The datasets used and/or analyzed during the current study are available from the corresponding author on reasonable request.

## List of symbols and abbreviations

*d*_*o*_/*d*_*i*_: Outer/inner diameter
ESI: Electro spray ionization
IBU: Ibuprofen
MBR: Membrane bioreactor
MF: Microfiltration
MWCO: Molecular weight cut off
NF: Nanofiltration
NSAIDs: Non-steroidal anti-inflammatory drugs
PhACs: Pharmaceuticals
UF: Ultrafiltration
UV: Ultraviolet
VIS: Visible
